# Bisphenols Threaten Male Reproductive Health via Testicular Cells

**DOI:** 10.3389/fendo.2020.00624

**Published:** 2020-09-11

**Authors:** Elikanah Olusayo Adegoke, Md Saidur Rahman, Myung-Geol Pang

**Affiliations:** Department of Animal Science and Technology and BET Research Institute, Chung-Ang University, Anseong, South Korea

**Keywords:** bisphenol, Sertoli cell, Leydig cell, germ cell, apoptosis, tight junction

## Abstract

Male reproductive function and health are largely dependent on the testes, which are strictly regulated by their major cell components, i. e., Sertoli, Leydig, and germ cells. Sertoli cells perform a crucial phagocytic function in addition to supporting the development of germ cells. Leydig cells produce hormones essential for male reproductive function, and germ cell quality is a key parameter for male fertility assessment. However, these cells have been identified as primary targets of endocrine disruptors, including bisphenols. Bisphenols are a category of man-made organic chemicals used to manufacture plastics, epoxy resins, and personal care products such as lipsticks, face makeup, and nail lacquers. Despite long-term uncertainty regarding their safety, bisphenols are still being used worldwide, especially bisphenol A. While considerable attention has been paid to the effects of bisphenols on health, current bisphenol-related reproductive health cases indicate that greater attention should be given to these chemicals. Bisphenols, especially bisphenol A, F, and S, have been reported to elicit various effects on testicular cells, including apoptosis, DNA damage, disruption of intercommunication among cells, mitochondrial damage, disruption of tight junctions, and arrest of proliferation, which threaten male reproductive health. In addition, bisphenols are xenoestrogens, which alter organs and cells functions via agonistic or antagonistic interplay with hormone receptors. In this review, we provide *in utero, in vivo*, and *in vitro* evidence that currently available brands of bisphenols impair male reproductive health through their action on testicular cells.

## Introduction

In recent years, the declining trend in male reproductive health has generated public concern, and industrialized countries are the most affected (1, 2). A recent study indicated that the fertility rate has drastically declined in the United States of America, European countries, Japan, South Korea, and Singapore (2). About half of these infertility occurrences are linked to male factors (3–5). Studies relating to infertility in both human and animals have identified endocrine disruptors, including bisphenols, among etiologies (2, 5, 6). Bisphenols are man-made organic chemicals used to manufacture plastics, epoxy resins, and other products. The most common and widely used analog of bisphenol, bisphenol A (BPA), was designed by Diani in 1891 and synthesized by Zincke in 1905 (7). However, there are growing concerns about BPA which constitutes a major component of food packaging, plastics and other household products becoming a threat owing to its tendency to leach into the surroundings. The perceived harmful tendency of BPA has led to a complete ban, regulatory policies, and search for safer substitutes in many countries (8–10). Consequently, there is variation in its usage in different countries (8, 9).

As BPA is being removed from consumer products, there is a progressive move to its derivatives: bisphenols F, E, B, and S as materials for polycarbonate resin (11). Other industrial application of BPS include wash fastening and electroplating (12), BPF epoxy resins are used in lacquers, liners, adhesives, dental sealants, and food packaging (13), while other analogs of BPA such as 2,2-bis-(3,5- dibromo-4-hydroxyphenyl)propane (TBBPA), are commonly used as fire retardant in several materials (11). An ideal substitute for replacing BPA, whose safety is of public concern, should be inert or less toxic. Unfortunately, these analogs have been implicated in male reproductive health problems and found in several household commodities, for instance; body cream, shampoo, meat, and milk (14, 15), making them imperfect substitutes for BPA. Bisphenols are ubiquitous contaminants in humans, livestock, wildlife, and the environment (16). Humans get exposed to bisphenols through food, skin, and inhalation (17, 18). Protective coatings of drinks and food cans, and household water containers are made of phenolic epoxy resins that contain BPA (8).

Once BPA and analogs are absorbed into the body, their major targets include testicular cells (19, 20). Functional cells of the testes include Leydig, Sertoli, and germ cells (21). Although the testis houses other important cells such as peritubular myoid, nerve, blood, and lymphatic endothelial cells, information on impact of bisphenols on these cells in relation to male reproductive health is few (22). Leydig cells produce testosterone, which perform a crucial function in differentiation of the germ cells and maintenance of testicular functions (21). Additionally, testosterone produced by Leydig cells perform important functions in the maintenance of the prostate gland (23). Sertoli cells phagocytize apoptotic germ cells to maintain testicular homeostasis for normal spermatogenesis and regulate germ cell proliferation and differentiation (24). These cells control male somatic sex determination during embryogenesis and spermatogenesis in adulthood (25). In addition, Sertoli cells secrete lactate and pyruvate, which are sources of energy for germ cells (26–29). Their number in the testes is, therefore, closely related to testicular volume and sperm yield (21). The production of viable spermatozoa involves a sequence of gradual differentiation of germ cells via mitosis and meiosis, and final transformation into mature sperm (30, 31).

Previous studies have revealed estrogen signaling as important signaling involved in endocrine disrupting activities of bisphenols, especially BPA (11, 17, 32). Estrogen signaling occurs through multiple pathways in which estrogen receptors (ERα and ERβ) regulate transcription of target genes directly or indirectly (33). BPA binds with cytoplasm estrogen receptors (cERs) or ERs located in the nucleus (nERs) in the genomic pathway. The binding to these receptors affects nuclear chromatin function and regulates the transcription/translation of genes and protein. Consequently, the cell proliferation, differentiation, and survival are altered (17, 34). In non-genomic signaling pathway, BPA could bind to G-protein coupled receptor (GPR30) on the membrane of testicular cells especially sperm cells (17). The activation of these receptors by BPA in sperm cells results in rapid phosphorylation of mitogen-activated protein kinase (MAPK), phosphatidylinositol 3-kinase (PI3K), protein kinase A (PKA), and alteration in levels of cyclic adenosine monophosphate, protein kinase C, and intracellular calcium which result in serious cellular effect (11). BPA can also elicit its effect on testicular cells via reactive oxygen species-mediated damage and apoptosis through activation of pro-apoptotic signaling (MAPK, Fas/FasL, Caspase 3 and 9, Bax) (35).

**Figure 1:**
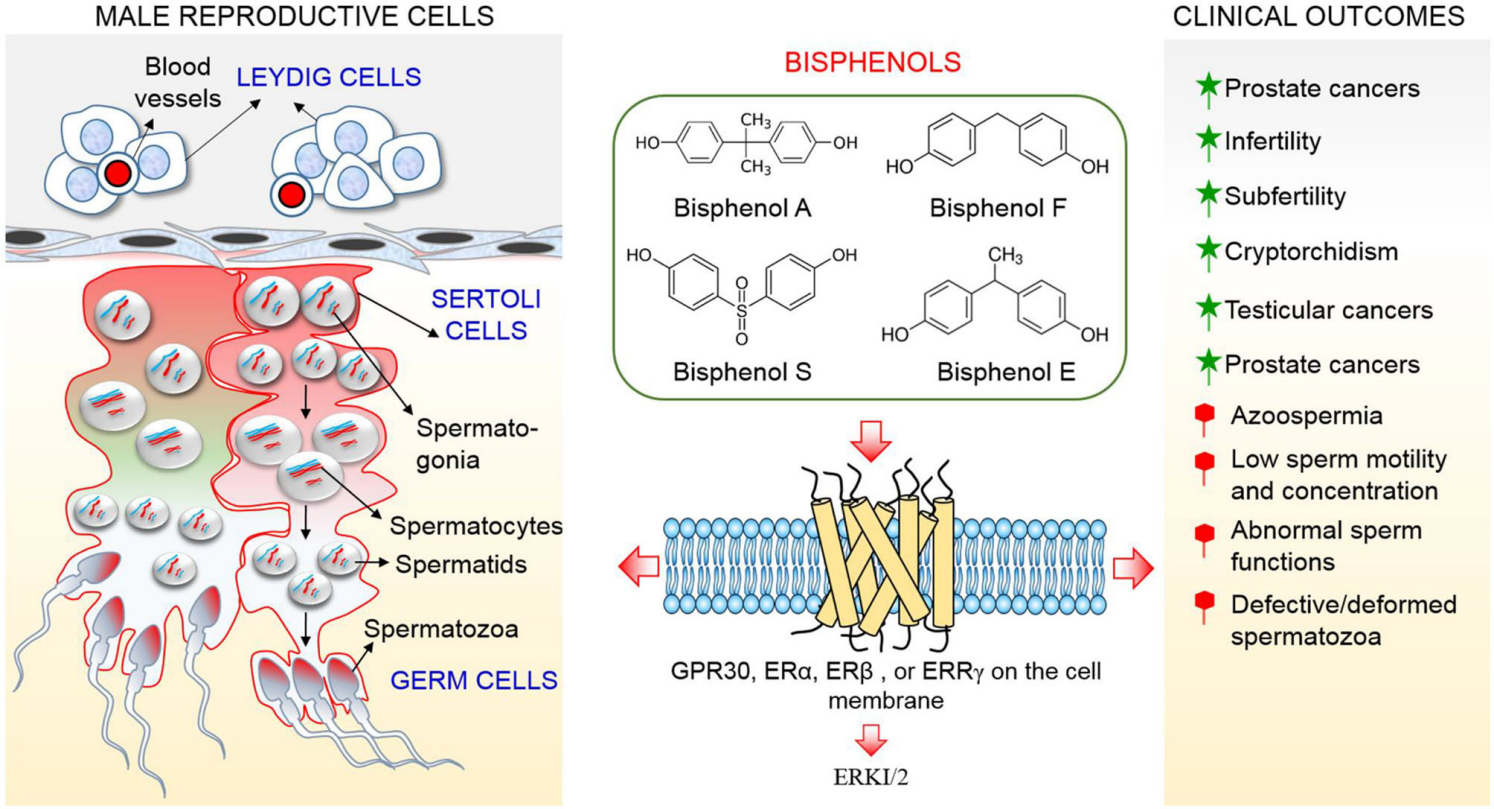
Schematic representation of the action mechanism of bisphenols on testicular cells.

Although, a study indicated that BPA probably incapable to elicit observable effects at low concentration through estrogen receptors (ERα and ERβ) by demonstrating a low affinity between BPA and these receptors (32), recent studies reported that BPA possesses a strong affinity with membrane-bound estrogen receptors and G protein–coupled receptor 30 (GPR30) and evokes cellular effects at low doses (picomolar and nanomolar concentrations), which are lower compared to concentrations needed to stimulate nuclear ERs (36, 37). This review suggests that extremely low doses of BPA that are incapable of producing detrimental effects in tissues and organs via ERα and ERβ will produce negative effects in testicular cells through GPR30 and ERR-γ which are more abundant in the cells (Figure 1). Therefore, the objective of this review is to clarify conflicting studies surrounding the effect of bisphenols on male reproductive health, and produce evidence that BPA and currently available analogs threaten male health and fertility through their action on testicular cells, which results in alterations to testicular functions and culminates in impairment of male reproductive health.

## Function of Leydig Cells in Male Reproductive Health

Leydig cells are found in the interstitial spaces of the testis (38). They are vital parts of male reproductive organ development and reproduction (39). Androgens produced by Leydig cells are essential for the differentiation of male genitalia and masculinization in response to luteinizing hormones from the pituitary (39). Sexual differentiation in males is a complex sequence of processes that involves activities of hormones produced by somatic cells in the gonads, including Leydig cells (38). There are two populations of Leydig cells in eutherian mammals: fetal and adult Leydig cells (38–40). The post-natal surge in androgens has led to suggestion that there is neonatal or infantile population of Leydig cells in certain species such as humans and primates (38, 40). The fetal Leydig cells are found in the embryonic testes following formation of the testis until parturition (40, 41). In mouse, the fetal Leydig cell secrete androstenedione which is consequently converted to testosterone by hydroxysteroid 17-beta dehydrogenase 3(HSD17B3) produced by Sertoli cells, whereas fetal Leydig cells of rat produce testosterone commencing from gestational day 15.5 (39–41). The adult Leydig cells synthesize testosterone needed for the development of male reproductive organ and the commencement of spermatogenesis (38). Although both fetal and adult Leydig cells possess some distinctions in morphological characteristics, they perform same function of androgen production (38). Androgens and other hormones produced by Leydig cells are indispensable to male reproductive system development and health (38). The absence or dysfunction of these cells give rise to maldevelopment of reproductive organs and disorders associated with incomplete masculinization of the male fetus (41, 42). Additionally, the alteration of Leydig cell function can adversely impair fertility in men (43). Normal male sex differentiation procedure involves movement of the testes from their initial location close to the kidneys into the extracorporeal position inside the scrotum. There are two separate and successive phases of testicular descent: the intraabdominal phase, where the testes migrate to the abdominal base; and the inguinoscrotal phase, which involves the movement of the testes through the inguinal canal into the scrotum (43). Each of these phases is controlled by specific hormones; insulin-like 3 (INSL3) and testosterone (40), produced by Leydig cells. The intraabdominal phase which occurs in man between 8 and 10 weeks of gestation is controlled by INSL3, while testosterone regulates the inguinoscrotal phase which occurs between 20 and 26 weeks of gestation (44, 45). Therefore, Leydig cells perform a critical role in male reproductive development and health.

## The Effects of Bisphenols on Leydig Cells and the Development of Male Reproductive Organs

Leydig cells are the main producers of testosterone in the male reproductive system, and harm to them can lead to infertility (6). A previous study identified a high occurrence of undescended testes in several developed nations (43); one third of male born prematurely have unilateral cryptorchidism, while 2–8% cases are found in full-term males, indicating cryptorchidism as prevalent male reproductive abnormality (46). Leydig cells have been identified among the target cells of bisphenols and other environmental contaminants (47–49). There is increasing evidence of the detrimental implication of BPA and derivatives on health and function of male reproductive system acting via Leydig cells in a dose-dependent manner (48, 49). A previous study examined the effect of BPA on testicular testosterone production using human (6th−11th gestational weeks) and rodent [(Wistar rat:14.5 dpc), (C57BL/6 mice 12.5 dpc)] testicular explants using Fetal Testis Assay (FeTA) method and found that testosterone production was unaffected when exposed to 10^−12^ M BPA for 3 days. However, a reduction in testosterone secretion was noticed with exposure to 10^−8^ M BPA (48). By implication, the effect of BPA on Leydig cells is dose dependent. In the same study, 10^−8^ M BPA decreased testosterone production in human testicular explant, while a higher concentration of 10^−5^ M was required to produce same effect in mice and rats. These results indicate that the effect of BPA is species dependent. Similar to the results obtained from testosterone secretion, BPA decreased mRNA levels of INSL3 in both human and mouse testicular explants in species-dependent manner (48). Meanwhile, a new experiment investigating the effect of BPA on Leydig cells using human and two strains of rodent testicular explants (49) reported that the administration of BPA doses of 10^−8^ M and 10^−5^ M (the concentration that decreased testosterone and INSL3 in humans in an earlier study) for 72 h suppressed testosterone secretion in Sprague-Dawley fetal rat testicular explants, while a higher concentration of 10^−5^ M repressed testosterone in Wistar strain. This report implies that higher concentration of BPA is required to reduce testosterone production in Wistar rat, while a lower concentration similar to that of human is required to produce same effect in Sprague–Dawley strain. Therefore, whether data regarding the effect of bisphenols on Leydig cells from all strains of rodents under the same conditions can be used to make inferences for human risk assessments requires further investigation. Another experiment conducted using the TM3 Leydig cell from mice indicated that BPA decreased testosterone production, cell viability, growth, metabolic active mitochondria, and induced cell death and alteration of mitochondria membrane potential (50). Exposure of adult Leydig cells to 0.01 nm BPA altered testosterone production by 25%, while 2.4 μg/kg/day decreased testosterone production, androgen biosynthesis, and *CYP17* gene expression in rats in the same study (6). Another study conducted to investigate the involvement of BPA in the maldevelopment of male reproductive organs and heath demonstrated that BPA increased aromatase mRNA levels but suppressed testosterone production in R2C cell line (from rats) *in vitro* (51).

Furthermore, studies on the effect of bisphenols on development of male reproductive organs revealed that 10,000 nmol/L of BPA and its analogs; BPS and BPF, reduced the mRNA level of fetal Leydig cell-related genes [Steroidogenic acute regulatory protein (*Star*), 3 beta-hydroxysteroid dehydrogenase/Delta 5–>4-isomerase type 1 (*Hsd3b1*), cytochrome P450 family 17 subfamily A member 1 (*Cyp17a1*), *INSL3*] in 12.5 dpc fetal mouse testicular explants cultured for 3 days (52). In the same study, the author expanded their earlier finding that used (FeTA) method (48) by adopting basal condition to investigate the differential effect of BPA on rat (14.5 dpc), mouse (12.5 dpc), and human (6.3–11.1 gestational weeks) testicular explants. It was confirmed that 1,000 nmol/L (10^−6^ M) of BPA significantly reduced basal testosterone secretion of human and mouse fetal testes (52). The authors concluded that the minimum observed adverse effect level is 100-fold higher in mouse than in human testes and 100 or 1,000-fold higher in rat than in human testes in basal conditions (52). Moreover, higher occurrences of undescended testes may stem from impairment of the functions of Leydig cells during embryonic stage. In another study, BPA concentration in the blood was negatively correlated with INSL3 expression level (53). Several other studies have indicated that BPA and its analogs reduced both Leydig cell number and testosterone production (54–59). Interestingly, a clinical study conducted on 160 neonate males (Control:80, hypospadias patients: 80) suffering from hypospadias had seven folds BPA concentration in their blood compared to normal newborns (60). Testosterone controls the masculinization of male genitourinary system and a decrease or alteration in its production between days 15 and 18 post-coitus results in developmental defects in male rat fetuses (52). Despite the variation in the mode of study (*in vitro* or *in vivo*), route (subcutaneous or gavage), duration (short or long), and species, all researchers confirmed the effect of bisphenols on Leydig cells. Variations observed in reports are linked with dosage, species, age, duration, and solvent used for dissolving bisphenols. Meanwhile, the interspecies discrepancies present critical concern since animal studies are commonly employed in risk assessment of bisphenols. Human risk assessment data extrapolation from *in vivo* animal studies has generated a concern because metabolic process of BPA in man and rodent is different (61–63). The sensitivity to BPA in rodent fetal and adult-type Leydig cells cannot be comparatively assessed because most studies involving mature animals were conducted *in vivo*, moreover, hypothalamus-pituitary-testicular axis can be affected by bisphenols at varying developmental stage differently (62). Furthermore, the effect of BPA and analogs could be cell specific, e.g., BPA, BPF, BPS, BPE, BPB, and bisphenol A diglycidyl ether (BADGE) inhibit testosterone production in Leydig cells from human testicular explants, whereas Sertoli and germ cells were not affected by the same concentration in the study (54). This suggests variation in the action mechanism of bisphenols on different cells of the testes. A summary of effects of bisphenols on Leydig cells is shown in Table 1.

**Table 1:** Effects of Bisphenols on Leydig cells.

**Chemical name**	**Dosage**	**Species**	**Effects**	**References**
BPA	2.4 μg/kg/day	Long-Evans rat	Decreased testosterone production (1.62 ± 0.16 ng/ml; vs. control, 2.52 ± 0.21)	(6)
			Decreased androgen biosynthesis	
			Suppression of CYP17 gene expression	
			Inhibition of testicular steroidogenesis	
BPA	0.01	90-day old Rat adult Leydig cell	Decreased testosterone biosynthesis by 25%	(6)
BPA	10, 25, and 50 μg/ml	TM3 cell line	Decreased testosterone secretion by 30.4, 69.2, 79.5 % for 10, 25, and 50 μg/ml, respectively	(47)
			Decreased viability	
BPB	10, 25, and 50 μg/ml	TM3 cell line	Decreased testosterone secretion by 41, 76.1, and 91% for 10, 25, and 50 μg/ml, respectively	(47)
BPS	10, 25, and 50 μg/ml	TM3 cell line	Decreased testosterone secretion by 8.8, 7, and 19.4% for 10, 25, and 50 μg/ml, respectively	(47)
BPF	25 and 50 μg/ml	TM3 cell line	Decreased testosterone secretion by 3.8 and 13.8% for 25 and 50 μg/ml, respectively	(47)
BPA	10^−8^ M	Human (6.5–10.5 gestational weeks) testicular explant	Decreased testosterone by 20% compared to control	(48)
			Reduced expression of INSL3 by 20% compared to control	
BPA	10^−5^ M	Wistar rat (14.5 dpc) testicular explant	Decreased testosterone by approximately 50 % on 3rd day of culture	(48)
			Reduced expression of INSL3 by approximately 20%	
BPA	10^−5^ M	Sprague-Dawley Rat (14.5 dpc) testicular explants	Inhibition of testosterone by 10^−5M^ BPA diluted in DMSO at all periods 24 h: 53%; 48 h: 40%; 72 h: 39%,	(49)
			Suppressed INSL3 by 76%	
BPA	10^−5^ M	Man (7–12 gestational week) testicular explants	Inhibition of testosterone by 10^−5^ BPA diluted in DMSO by 28%	(49)
BPA	1, 10, and 100 μM	TM3 cell line	Decreased testosterone production by 22%, 28%, and 39%, for 1, 10, and 100 μM, respectively, when compared to the negative control	(50)
			Decreased cell viability	
			Decreased cell growth	
			Decreased metabolically active mitochondria	
			Alteration of mitochondrial membrane potential	
BPA, BPS, and BPF	10,000 nmol/L	Mice (12.5 dpc)	Reduced INSL3 expression	(52)
			Reduced expression of testosterone biosynthesis related genes (*Star, Hsd3b1, Cyp17a1*) and *Lhcgr*	
BPA, BPS, and BPF	10 nmol/L	Human (6.3–11.1 gestational weeks)	Decreased basal testosterone secretion	(52)
BPA and BPB	10^−9^-10^−5^ M	Human (46.7 ± 4.65) testicular explant	Inhibition of testosterone production (BPA, 28.7 and 39.2 % at 24 and 48 h, respectively) (BPB, 17 and 47% at 24 and 48 h, respectively.	(54)
BPAF	200 mg/kg/day	7 weeks Sprague–Dawley rat	Reduction of testosterone production by 90.6% compared to control	(55)
			Altered testosterone biosynthesis	
BPA	100 and 200 mg/kg/day	Wistar/ST rat	Reduced plasma and testicular testosterone production	(56)
			Reduced number of Leydig cell	
BPA	4, 40, and 400 mg/kg	Sprague–Dawley rats (Gestational day 21)	Disruption of fetal Leydig cell number, proliferation and distribution	(57)
			Downregulation of Leydig cell genes	
			Decreased expression of INSL3	

## Effect of Bisphenols on Leydig Cells and Testicular Dysgenesis Syndrome

**Figure 2:**
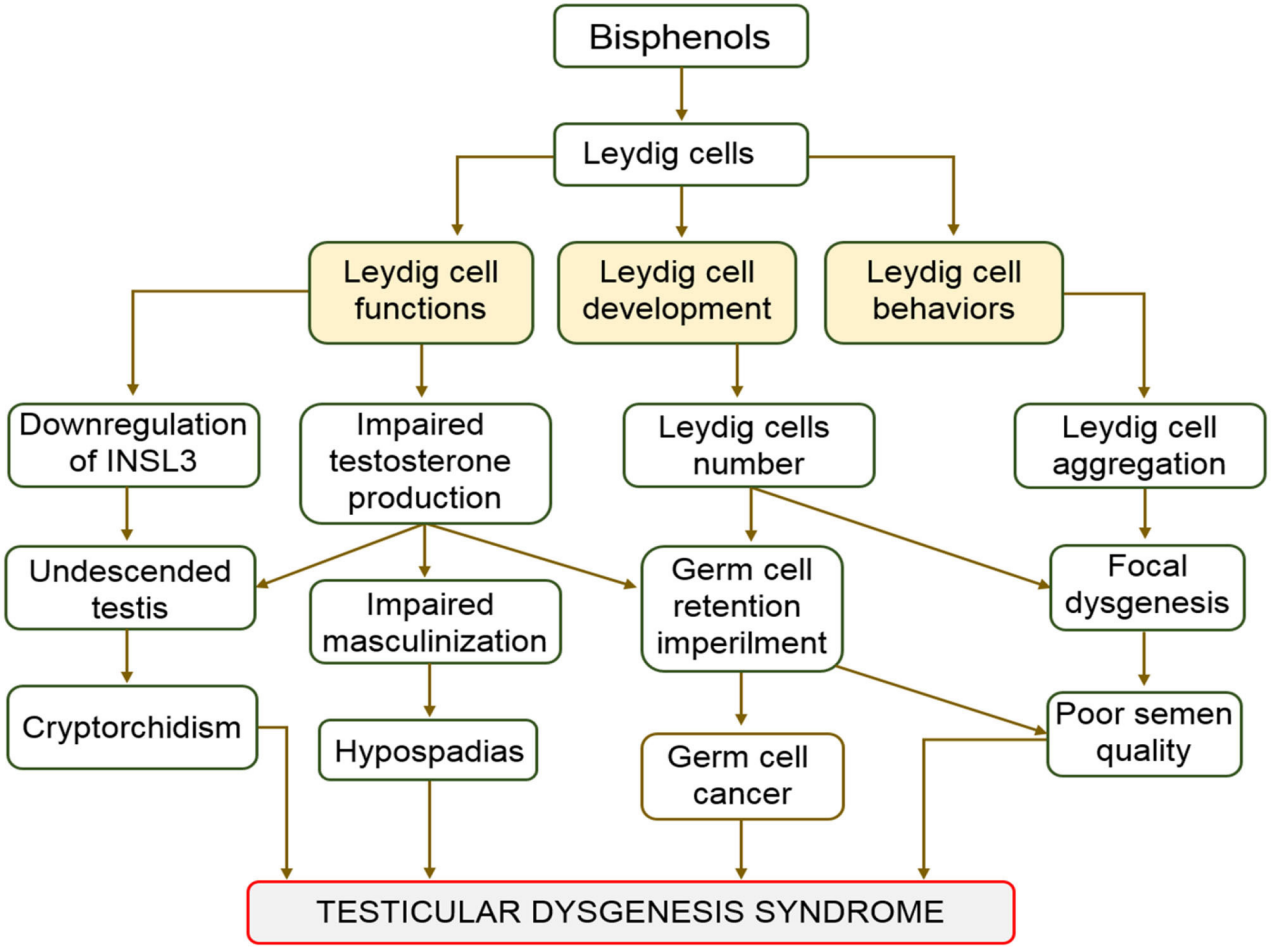
Schematic diagram of the effect of Bisphenols on Leydig cells.

Hypospadias, cryptorchidism, impaired spermatogenesis, and testicular cancer are categorized as testicular dysgenesis syndrome (TDS). They also represent indices of impaired prenatal testicular development (64, 65). This syndrome (TDS) is associated with embryonic maldevelopment of the testis, which impair differentiation of somatic cells (66). Cryptorchid testes mostly harbor twisted tubules and undifferentiated Sertoli cells (66, 67). Although many factors have been hypothesized as causes of TDS, some studies have linked TDS to the effect of endocrine disruptors on Leydig cells (68–70). Bisphenols are endocrine disruptors that can cause malfunctioning of Leydig cells (47–49). Cases of TDS are characterized by the failure of gonads to fully develop and emergence of intersex genitalia (64). The influence of BPA on human fertility and its involvement in several reproductive complications, including TDS, germ cell cancers, and Sertoli cell only syndrome have been reported (10, 71–73). In a study conducted to evaluate the BPA in the blood samples of 98 cryptorchid males admitted for surgery, serum BPA levels ranged from 4.1 to 89.8 ng/mL (74). The study concluded that serum BPA was positively correlated with cryptorchidism. Effect of BPA on Leydig cells resulting to hypoplasia of the cell can be complete or incomplete. Complete hypoplasia is characterized by presence of both male and female copulatory organs. However, atrophy or hypertrophy of testicles characterized incomplete forms (48). These findings prove beyond doubt the involvement of bisphenols, especially BPA in the maldevelopment of the male reproductive organs and system. The aftermath of decreases in the expression of INSL3 and testosterone synthesis are masculinization defects. Low fertility and germ cell cancer are major complications linked with testicular dysgenesis (43). The association between the actions of bisphenols on Leydig cells and testicular dysgenesis is as shown in Figure 2.

## The Action of Bisphenols on Leydig Cells and Prostate Health

Testosterone performs a very important function in male reproductive health. Reduction or alteration of its synthesis is connected to complications of hypogonadism and impairment of male reproductive health (52). Estrogen and testosterone execute crucial roles in the onset, advancement, and growth of prostate cancer (75). The growth and maintenance of prostate gland is regulated by testosterone and prolactin (23). Citrate production by prostate glands is performed by the highly specialized citrate-producing acini epithelial cells (23). The capability of the acini epithelial cells to produce substantial citrate depends on their zinc accumulating ability and inhibition of citrate oxidation. Meanwhile, the zinc and citrate levels are controlled by testosterone and prolactin (76, 77). There is increasing concern that exposure to xenoestrogen, including bisphenols, during critical developmental window may multiply vulnerability to prostate cancer. Leydig cells are involved in testosterone production and are major targets of bisphenols (47–49). Earlier research findings revealed that BPA decreases testosterone synthesis via Leydig cells, indicating that it is also a threat to prostate health. As shown in previous studies, BPA contamination is related to development of prostate cancer and elevates centrosome amplification *in vitro* (78). In another study, BPA caused enlargement of prostate and increased EGFR mRNA level in mature Sprague–Dawley rats when administered orally (79). Another study demonstrated that BPA enhanced human prostate stem cell proliferation (80). However, a recent study conducted by the same author using a rat model and human prostate epithelial cells indicated that BPA alone did not drive prostate pathology but low doses of BPA augment vulnerability to prostate cancer and induced homeostatic imbalance in adult prostate stem cell (81). At present, these studies indicate bisphenols, especially BPA, as predisposing factors of prostate cancer via decreased testosterone production. However, further investigation into the severity of the involvement of bisphenols as a single chemical and in a mixture with other xenoestrogen in the development of prostate cancer is required.

## Action Mechanisms of Bisphenols on Leydig Cells

As bisphenols are estrogenic in nature, estrogen receptors (ERs) are expressed in Leydig cells and are controlled by estrogen activities (82). Information regarding the localization of ERs in Leydig cells has been inconsistent, they were reported absent (83) and present (84) in the mouse Leydig cell. It was found to be localized in fetal rat Leydig cells (85), but not in adult rat Leydig cells (86). Similarly, ERs were not detected in mouse Leydig cells (6). It was further demonstrated that ERα expression is non-detectable in human fetal testes (87), suggesting that there is no involvement of ERα in impact of BPA on human testes. This result was considered applicable in mice because BPA induced decrease in testosterone production was maintained following ERα withdrawal (48). Meanwhile, studies regarding the presence or absence of ERβ in human and mouse Leydig cells need to be confirmed in future research. Low concentrations of BPA have reportedly elicited effects via GPR30 or estrogen-related receptor gamma (ERR-γ) (88–90). Both GPR30 and ERR-gamma were expressed in human and mouse fetal testes (48) and ERR-γ has a high affinity for BPA (91). Therefore, GPR30 and ERR-γ represent means by which bisphenols act on testicular cells.

## Bisphenols, Sertoli Cells, and Male Reproductive Health

### Function of Sertoli Cells in Spermatogenesis

Spermatogenesis is a successive development of male germ cells to mature spermatozoa. It involves mitotic and meiotic divisions of germ cells. Sertoli cells play a key role in every stage of spermatogenesis, by clinching tightly to developing germ cells in the seminiferous tubules thereby providing a suitable milieu necessary for their development. Sertoli cells produce pyruvate for nourishment of germ cell. Lactate and pyruvate produced by Sertoli cells are needed by germ cells for energy and survival (26–29), thereby providing nutrition for their development (92). About thirty to fifty germ cells at various developmental phases can be nourished in the seminiferous epithelium by every Sertoli cell (93). The cumulative number of Sertoli cell is positively correlated with testicular size and sperm count (94). Tissue transformation that takes place during spermiation is achieved through the activities of proteases produced by Sertoli cells (21). Similarly, plasminogen activator which facilitates the migration of preleptotene spermatocytes are produced by Sertoli cells (21). Importantly, Sertoli cells expansive junctional networks and communication provide structural support for developing germ cells (20).

### Effect of Bisphenols on the Blood-Testis Barrier and Its Implication on Spermatogenesis

Several studies have provided strong evidence that BPA derivatives (BPE and BPS) affect Sertoli cell functions (19, 20, 95). An experimental study on the effect of BPA on Sertoli cells at >150 μM concentration time and dose dependently reduced cell viability, while those exposed to 200 μM BPA reduced to approximately two-thirds of the control (96). The study further revealed that Sertoli cells treated with BPA *in vitro* at a concentration of 200 μM induced morphological distortions such as collapse of cytoskeleton, chromatin impairment, and DNA damage in the cells. Immunocytochemistry studies of the cells showed the expression of caspase-3, colocalization of active caspase-3, and fragmentation of actin filaments (96). The authors concluded that stimulation of apoptotic pathways within the cells rather than necrosis was responsible for their death (96). In another study involving Sertoli cells, BPA induced cellular damage and apoptosis; the BPA-induced damage was attributed to its ability to block endoplasmic reticulum-Ca^2+^ homeostasis (97). BPA was also reported to affect anchoring junction which attached spermatids to Sertoli cells (98). This effect of BPA characterized the human Sertoli cell only (SCO) testes, in which the testes were void of blood-testis barrier and constituent proteins, especially connexin 26, which mediates adhesion or communication at the site of attachment of Sertoli cells and spermatogonial are downregulated (99). Several other studies (19, 20, 42) showed that bisphenols, especially BPA, impair male reproductive health. BPA reportedly perturbed Sertoli cell tight junction, downregulated the level of blood-testis barrier constituent proteins (JAM-A, ZO-1, N-cadherin, connexin 43), activated ERK1/2, and redistributed cell-cell interface proteins (19, 20). Although these effects were reported to be non-significant in adult rats, significant effects were observed in immature (20-day-old) rats at the same concentration, indicating a higher susceptibility of immature rats and infants to BPA (19). Information regarding the effect of bisphenols on Sertoli cells isolated from adult rats is not available. Therefore, it remains uncertain whether the effect will be significant if Sertoli cells isolated from mature rats are exposed to BPA *in vitro*. When cultured *in vitro*, Sertoli cells form a blood-testis barrier and intercellular junctions that mimic *in vivo* conditions between 48 and 72 h after culture (100, 101). Using *in vitro* methods, another study (101) investigated the mechanism of BPA action on Sertoli cells and confirmed that BPA interferes with junctional proteins of the cells, for example; occludin, connexin 43, and E-cadherin. In addition to *in vitro* evidence, *in vivo* studies involving 6-week-old male mice revealed that BPA downregulated a wide range of genes connected to Sertoli cell function [Musashi RNA Binding Protein 1 (*Msi1h*), Nuclear Receptor Coactivator 1(*Ncoa1*), nidogen 1 (*Nid1*), Heat shock protein beta-2 (*Hspb2*), and GATA-binding factor 6 (*Gata6*)] following prenatal exposure (102). Downregulation of the junctional and functional proteins of Sertoli cells is potentially capable of disrupting the blood-testis barrier, thereby impairing spermatogenesis (103). Clinical and laboratory reports indicate that BPA exerts higher effects on male reproduction and fertility after prenatal and neonatal exposure than adults because they are resistant to BPA (19, 104, 105). For example, oral administration of 0.02–50 mg/kgbw doses of BPA to adult rats did not alter normal sperm production; meanwhile, it disrupted the blood-testis barrier integrity when neonatal rats were treated with 50 mg/kgbw/day of BPA. Similar result was obtained during *in vitro* BPA treatment of rat Sertoli cells at 40–200 μM (76). Most effects of bisphenols on testicular cells become visible after many years of accumulated effects at a cellular level. A summary of the effect of bisphenols on Sertoli cells is shown in Table 2.

**Table 2:** Effects of bisphenols on Sertoli cells.

**Chemical name**	**Dosage**	**Species**	**Effects**	**References**
BPA	50 mg/kg (Rats)	Sprague–Dawley rats and Wistar rats (20 day old);	Disruption of the blood-testis barrier integrity	(19)
BPA	200 μM (Sertoli cell)	Sertoli cells (20-day-old Sprague-Dawley rats)	Perturbation of Sertoli cell tight junction permeability barrier	(19)
			Activation of ERK1/2 in the cell	
			Downregulation of basal ectoplasmic specialization and gap junction at the blood-testis barrier	
BPA	150–200 μM	18-day-old Wistar rats Sertoli cell	Decreased cell viability	(96)
			Induction of membrane blebs, cell rounding, cytoskeletal collapse, chromatin condensation, and DNA fragmentation	
			Expression of caspase-3	
			Disorganization of the actin cytoskeleton	
			Decreased hormone (transferrin) secretion	
BPA	200 μM	Mouse Sertoli TTE3 cells	Induction of cellular damage and apoptosis	(97)
			Induction of endoplasmic reticulum stress	
			Endoplasmic reticulum Ca^2+^ homeostasis blockage	
BPA	20 and 200 μg/kg	ICR mice (3 months old)	Impairment of ectoplasmic specialization between the Sertoli cell and spermatids	(98)
		Wistar rat (4 months)	Incomplete, redundant ectopic specialization	
BPA	200 μM	Rat and SerW3 Sertoli cell line	Perturbation of the Sertoli cell tight junction permeability barrier function	(99)
			Downregulation of blood-testis barrier proteins	
			Redistribution of blood-testis barrier-associated proteins	
			Alteration of the distribution of integral membrane proteins and their peripheral adaptors	
BPA	45 μM	SerW3 Sertoli cells	Alterations of Sertoli cell functions	(101)
			Metabolic, endocrine and/or paracrine dysfunctions	
BPA	50 mg/kg	ICR mice (6 weeks)	Downregulation of Sertoli cell-related genes (*Msi1h, Ncoa1, Nid1, Hspb2*, and *Gata6*)	(102)
BPA	2.4 μg/kg/day	Neonatal Holtzman rat	Impairment of fertility	(105)
			Perturbations tight junctions and decreased expression of junctional proteins	
BPA	40 and 200 μM	Human Sertoli cell (12, 23, and 36-year old)	Truncation and depolymerization of actin	(106)
			Microfilaments	
			Disorganization of F-actin	
			Changes in the localization and distribution of F-actin regulatory proteins in Sertoli cell epithelia	
			Retraction of actin microfilaments	
BPA	4, 40, and 400 mg/kg	Sprague–Dawley rat	Downregulation of Sertoli cell genes	(57)

### Bisphenol Effects on Sertoli Cells and Testicular Homeostasis

**Figure 3:**
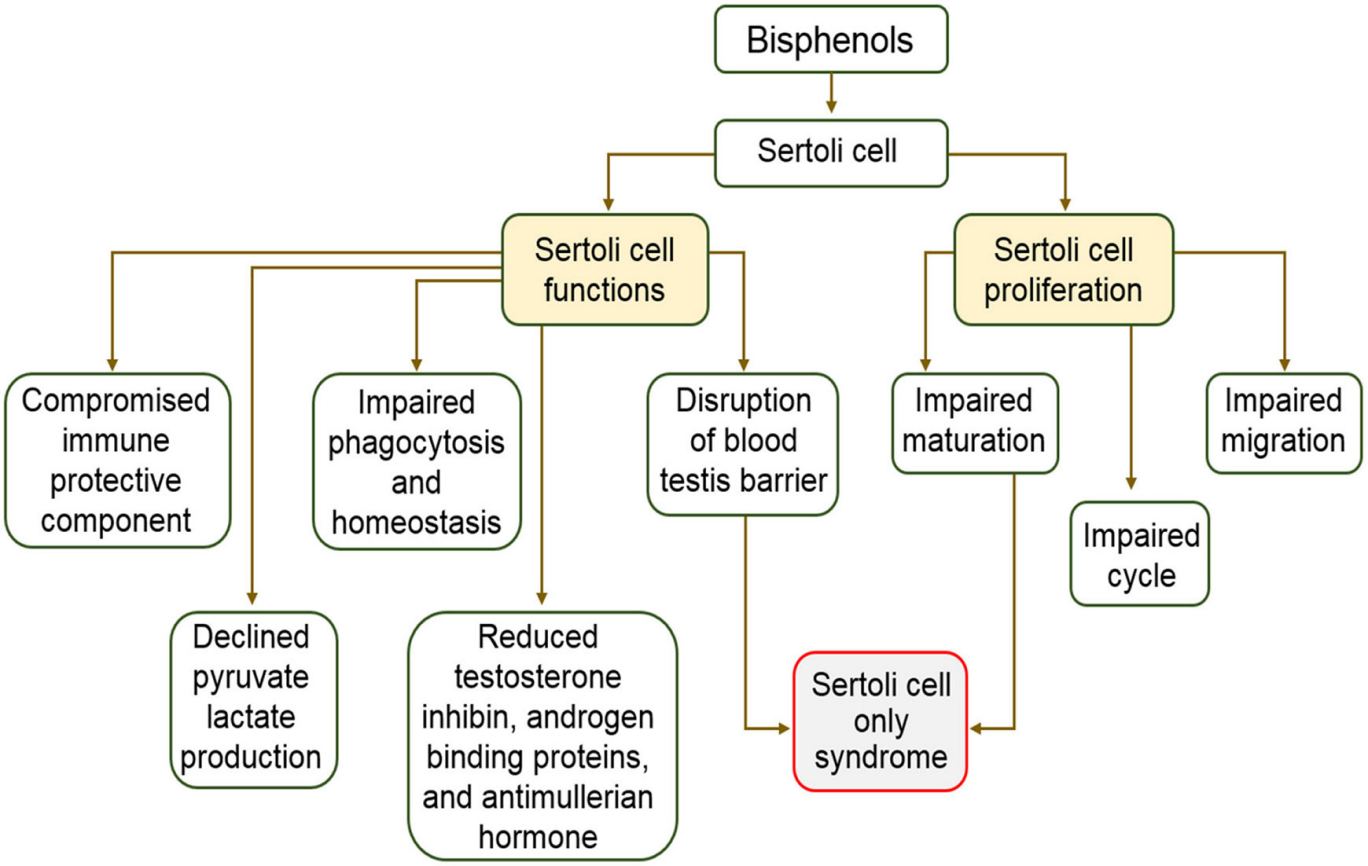
Schematic description of the effects of bisphenols on Sertoli cells and male reproductive health.

During spermatogenesis, few developing germ cells experience programmed cell death while some exfoliate some cytoplasmic materials following completion of differentiation procedures (107). Phagocytosis activities of Sertoli cells rid the testes of dead germ cells and exfoliated materials from full developed germ cells. Sertoli cells ensure elimination of noxious materials originating from dead cells and the removal of autoantigens that may induce an autoimmune response in the testes. Alteration of Sertoli cell phagocytosis is responsible for disease development and testicular dysfunction, consequently, infertility. In addition, disruption of phagocytic function of Sertoli cells interferes with spermatogenic cycle (108). Critical cellular functions like phagocytosis, structural support, and movement are achieved via actin cytoskeleton. Meanwhile, research findings on the effects of BPA on the actin cytoskeleton of the Sertoli cells revealed that BPA induced changes in the organization and location of F-actin proteins in Sertoli cell epithelia (106). The distribution of F-actin system was dose dependently disorganized when human Sertoli cells were cultured in the presence of BPA *in vitro* (106). The same study found that 0.4 μM BPA caused reduction of Sertoli cells actin microfilament. However, high doses, ranging from 40 to 200 μM, of BPA retracted actin microfilaments close to the nucleus (106). BPA also caused disorganization of the actin cytoskeleton in rat Sertoli cells (96). These changes were attributed to the mislocalization of two actin regulatory proteins leading to its failure to aid Sertoli cell blood-testis barrier function (17, 106). In addition, another recent study revealed that disengagement of spermatozoa from Sertoli cells at spermiation is controlled by modifications in arrangement of actin cytoskeletons at the apical ectoplasmic specialization (109). The disruption and mislocalization of actin proteins did not only interfere with testicular homeostasis but lead to untimely release of spermatids into the seminiferous epithelium and are consequently trapped within seminiferous epithelium. These studies confirm that through the action of BPA on actin cytoskeleton, Sertoli cell phagocytic function and testicular homeostasis are impaired. The description of the effects of bisphenols on Sertoli cells and male reproductive health is represented in Figure 3.

### The Action of Bisphenols on Sertoli Cells and the Defense of Testicular Immunity

Developing germ cells within the seminiferous epithelium are shielded by Sertoli cells being immune privileged cells (110). They perform this critical role by secreting chemicals that can suppress stimulation of pro-inflammatory cytokines and growth of B and T lymphocytes (111). Previous studies show that they synthesize complement inhibitors (112), and endured exposure to antigen (113). In addition, they secrete several protective factors that perform a critical function in immunomodulation and protection of spermatogonia and spermatids (111, 112, 114). Moreover, galectin-1, a highly conserved β-galactosidase-binding protein, capable of inhibiting pro-inflammatory cytokine activation was also discovered in Sertoli cells (115). Sertoli cells are a primary target of BPA and its analogs BPE and BPS impair its functions (19, 20, 95). The impairment of Sertoli cells defensive and immune functions is highly critical to the testes and male reproductive health because it predisposes developing germ cells to external attack. Despite varying dosages and strains of animals used in investigating the effects of bisphenols on Sertoli cells, all studies confirm the negative effect of bisphenols on Sertoli cells, especially during the neonatal window of development.

### Action Mechanism of the Effects of Bisphenol on Sertoli Cells

Bisphenols are endocrine disruptors that elicit their impact via affinity with estrogen, androgen, or thyroid hormone receptors (88). These steroid receptors are expressed in Sertoli cells (116, 117). The exposure of fetal rats to BPA reportedly activated Raf1 and p-ERK1/2 in the testes while further evaluation indicated increased level of Raf1 and ERK1/2 proteins in Sertoli cells in response to BPA exposure (118). A study also established that disruption of Sertoli cell tight junction barrier by BPA was accompanied by upregulation of p-ERK in Sertoli cells (19). Another study demonstrated that upregulation of pERK1/2 in cultured Sertoli cells with established junctional barrier declined to lowest level following BPA withdrawal, indicating the involvement of ERK1/2 in BPA induced disruption of Sertoli cell tight junction barrier (119). Although PD98059, an inhibitor of ERK, suppresses the BPA-induced ERK1/2 upregulation in Sertoli cells (120), whether the same inhibitor can repress other effects of BPA on rat and human Sertoli cells remains unknown. Information regarding the mechanism of BPA analogs on Sertoli cells is lacking. Therefore, studies regarding whether BPA analogs have the same or different mechanisms of action on Sertoli cells is necessary. Nonetheless, these findings indicated that BPA elicits its effects on Sertoli cells via the estrogen-ERK signaling pathway.

### Bisphenol Effects on Germ Cells and Spermatogenesis

Differentiation process by which spermatogonial stem cells become full developed spermatozoa is called Spermatogenesis (121) Spermatogonial stem cells experience mitotic and meiotic cell divisions to become functional spermatozoa (121). The quality of germ cells largely determines the fertility of males. Meanwhile, research findings have shown the negative impacts of bisphenols on different growth stages of male germ cells (121–124). BPA concentrations of 10 and 100 μM reportedly induced apoptosis, meiotic abnormalities, and altered stemness properties of ICR (CD-1) and C57BL/6-TG-EGFP mice spermatogonial stem cells cultured for 1 week (121). In the same study, BPA inhibited proliferation and induced alterations in testicular germ cells. Similar findings were reported in another study wherein BPA decreased the density and survival rate of rat spermatozoa (122). Contrary to earlier reports (19) that bisphenols only elicit effects on neonatal males, it was demonstrated in a more recent finding that BPA caused DNA damage, reduced sperm count, and motility in adult rats (123). This study was also supported by a recent finding that BPA through IFNβ-XAF1-XIAP signaling pathway caused germ cell apoptosis in adult mice (125). The incidence of hypomethylation was discovered in the spermatozoa of neonatal males exposed to 2.4 μg of BPA/ pup. Another recent study found that BPA, BPE, and BPS at concentrations of 0.5, 20, and 50 μg/kg/day, respectively, induced oxidative stress and apoptosis, altered the transition of germ cell stages (1–VI, VII, and VIII), caused spermatogenic defects, and decreased sperm motility (126). The study further used *in utero* exposure to confirm that lower concentration of BPE and BPS (between calculated human exposure and no adverse effect doses of BPA) are sufficient to interrupt germ cell differentiation in males. By implication, BPE and BPS are not safe alternatives to BPA in terms of the threat they pose to male reproductive health. The same authors also reported in their earlier study that BPA (10 mg/kgbw), BPE (50 μg/kgbw), and BPS (10 mg/kgbw) caused developmental distortion during spermatogenesis, disrupted male germ cell differentiation, induced germ cell apoptosis and DNA breaks in pachytene spermatocytes in mice (127). BPA, BPB, BPF, and BPS at dosages of 50 μg/L abated the number of germ cells (spermatocytes and spermatids), reduced sperm motility, and daily sperm production (128). This condition represents a critical situation in male reproductive health. In addition, 2 and 20 μg/kgbw of BPA induced oxidative stress in epididymal spermatozoa, caused abnormalities in sperm morphology, and decreased epididymal sperm counts and motility (129). When mice were administered 50 mg/kg/day of BPA, the seminiferous tubule contained a lower number of germ cells and undifferentiated germ cells (130). BPS at a dosage of 50 μg/L induced the generation of reactive oxygen species (ROS) and the apoptosis in germ cells (131). Another *in vivo* study that investigated the effect of 1, 5, and 100 mg/kg body weight of BPA in rats observed absence of germ cells within seminiferous tubules. In addition, the seminiferous epithelia appeared to disintegrate and germ cells were disengaged from Sertoli cells. There were no germ cells in the epididymis but filled with cellular debris (132). In another study that bordered on the impact of BPA on male germ cells using chickens, the results showed that male chickens orally administered BPA dosage of 2 mg/kg body weight every 2 days for 23 weeks had smaller seminiferous tubules exhibiting constrained spermatogenesis (133). The induction of ROS, undifferentiated germ cells, and empty epididymal tubules are indicators of impaired spermatogenesis. These alterations attributed to BPA and its analogs are not only impairments of spermatogenesis but also represent threats to male reproductive health. The effects of bisphenols on germ cells are summarized in Table 3.

**Table 3:** Effect of bisphenols on germ cells.

**Chemical name**	**Dosage**	**Species**	**Effects**	**References**
BPA	100 μM	ICR mice	Alteration of motility characteristics, acrosome reaction, fertilization, and early embryonic development	(71)
			Downregulation of fertility-related proteins	
			Altered capacitation status	
BPA	10 and 100 μM	Germ cell (ICR mice)	Induction of apoptosis in cultured spermatogonial stem cells	(121)
			Inhibition of testicular germ cell proliferation	
			Alteration of stemness properties of spermatogonial stem cells	
			Induction of meiotic abnormalities in spermatogonial stem cells	
			Induction of proteome alterations in germ cells	
BPA	50, 100, and 200 mg/kg/day	Wistar male rats (aged 28 days)	Sperm abnormality	(122)
			Decreased sperm density and survival rate	
BPA	5.0 mg/kgbw	Holtzman rat (8 weeks)	Increased sperm DNA damage	(123)
			Decreased motility	
			Decreased sperm count	
BPA	2.4 μg/pup	Holtzman rat	Induction of hypomethylation	(124)
BPA	30 mg/kg/day	Kunming mice (8 weeks)	Induction of apoptosis in germ cells	(125)
BPA, BPE, and BPS	0.5, 20, or 50 μg/kg/day	CD-1 mice (Post-natal day 12 and 16)	Disrupted progression of germ cell development	(126)
			Decreased sperm motility	
			Induction of oxidative stress and apoptosis of germ cells	
			Spermatogenic defect	
BPA (10 mg/kgbw)	50 or 10 mg/kgbw	CD-1 mice (5–6 weeks)	Meiotic errors during spermatogenesis	(127)
			Reduced sperm production and quality	
			Disrupted male germ cell development	
BPE (50 μg/kgbw)			Induction of germ cell apoptosis and DNA breaks in pachytene spermatocytes	
BPS (10 mg/kgbw			Delayed cycle in germ cell development	
BPA, BPB, BPF, and BPS	50 μg/L	Rat (22 day old)	Reduced sperm motility	(128)
			Reduced daily sperm production	
			Reduced number of epididymal sperm	
BPA	2 and 20 mg/kgbw	Wistar rat	Abnormalities in sperm morphology	(129)
			Decreased epididymal sperm counts and motility	
			Induction of oxidative stress in epididymal sperm	
BPA	50 mg/kg/day)	FXRα^−1−^ mice	Reduced number of germ cells	(130)
BPS	50 μg/L	Sprague Dawley rats (70–80 days)	Generation of reactive oxygen species (ROS)	(131)
			Induction of apoptosis	
			Reduced number of germ cells	
BPA	1, 5, and 100 mg/kgbw	Sprague Dawley rat (Postnatal day 21)	Undifferentiated germ cells	(132)
			Empty epididymal tubules	
			Sloughing of germ cells	
			Altered germ cell maturity	
BPA	2 mg/kgbw	Chicken (white leghorn)	Constrained spermatogenesis	(133)
BPA	5 mg/kg/day	ICR mice (4 weeks)	Lower seminiferous tubule and mature spermatids	(134)
			Disruption of spermatogenesis	
BPA	1.2 and 2.4 μg/kg/day	Holtzman mice (Postnatal day 75)	Increased time taken for copulation	(135)
			Degeneration of the germ cell	
			Sertoli cell only syndrome	
			Sloughing of germ cells	
BPA	100 μM	ICR mice	Decreased number of motile sperm	(136)
			Altered spermatozoa mitochondria activities	
BPA	50 mg/kg bw/day)	ICR mice (8 weeks old)	Alteration of capacitated spermatozoa function and the proteomic profile	(137)
			Compromised fertilization capabilities of Spermatozoa	

### The Effects of Bisphenols on Sperm Functions

**Figure 4:**
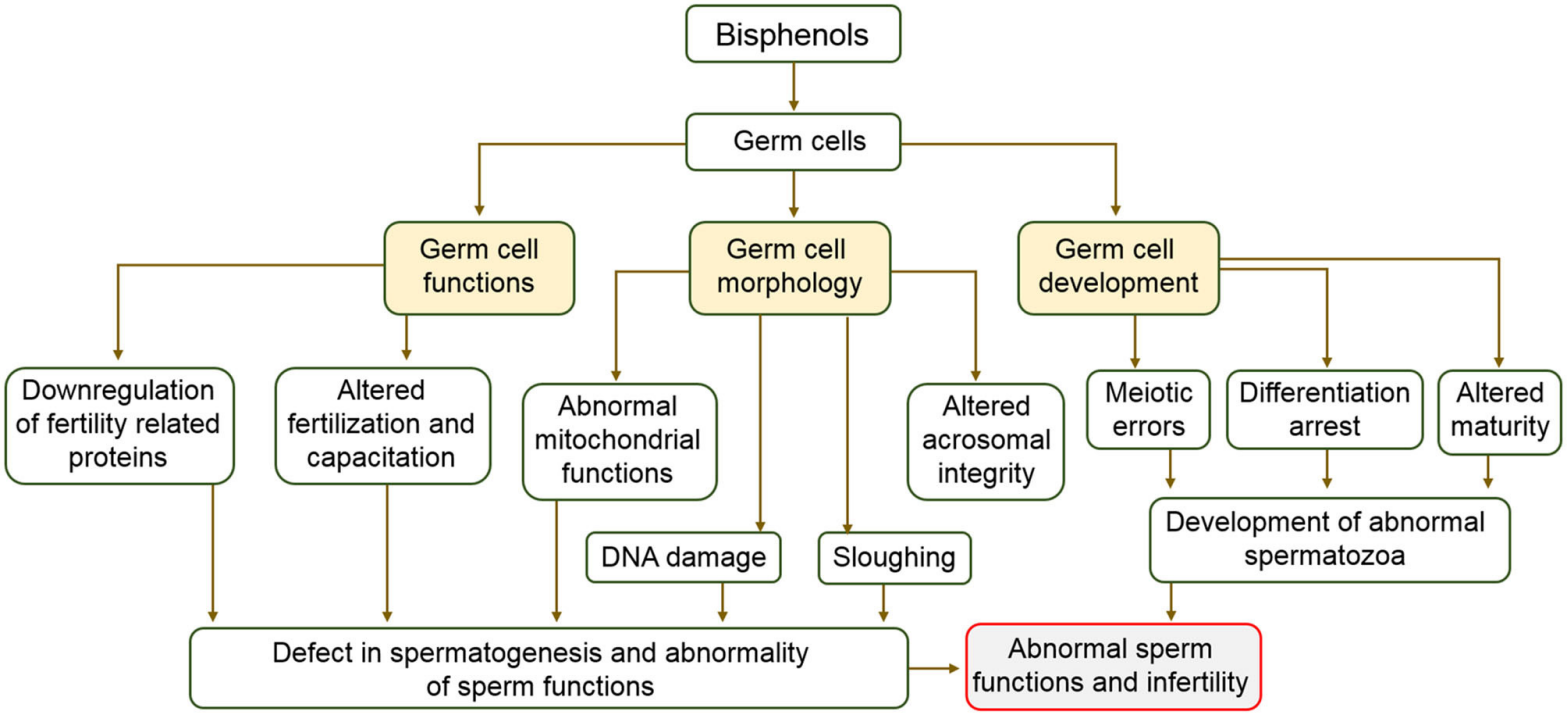
Schematic description of the effects of bisphenols on germ cell and male fertility.

Clinical data consistently revealed an adverse association between BPA exposure and sperm function. Decreased sperm counts and motility observed in occupationally exposed men (138) and infertile patients (139) positively correlated with their urinary BPA concentration. Additionally, an investigation involving middle aged men in Denmark showed lower sperm motility in persons in the upper percentile of urinary BPA concentration compared to those in lower percentile (140). Animal studies on impact of prenatal or neonatal exposure to BPA on spermatozoa showed deleterious aftermath on sperm production in adulthood. For example, the seminiferous tubule of ICR mice and Holtzman rats exposed to low concentration of BPA *in utero* contained reduced number of elongated spermatids and reduced sperm counts (134). The time taken for copulation in F1, F2, and F3 generations of the male offspring of rats exposed to 1.2 and 2.4 μg of BPA was significantly higher compared to that of their control counterparts in respective generation (135). Sperm motility, viability, mitochondrial functions, and intracellular ATP levels have been reported to be negatively affected by BPA through activation of the mitogen-activated protein kinase, phosphatidylinositol 3-kinase, and protein kinase-A pathways (136). A study which investigated the effect of BPA on sperm function revealed that *in utero* exposure of male vesper mice to BPA at 40, 80, and 200 μg/kg/day altered sperm membrane integrity and motility (141). In addition, pubertal exposure of C57BL/6J male mice to BPA at 50 mg/kg/day concentration caused deformity in ~9% of sperm population compared to the control group (142). The sperm acrosome integrity of postnatal day 50 male Wistar rats exposed to 5 and 25 mg/kg/day of BPA decreased by 8 and 16%, respectively (72). Similarly, same concentrations reduced sperm plasma membrane integrity by 2% (72). Capacitation-associated proteins in spermatozoa relates to male fertility (143, 144), unfortunately, these proteins are downregulated by BPA (145). Maternal transfer of BPA during nursing was reported caused impairment of spermatozoa in male offspring (146). BPA concentrations of 5 and 50 mg/kg/day reduced sperm motility and intracellular ATP levels of ICR mice (147). In another experiment in which the effect of BPA on sperm function was investigated, BPA concentrations of 50 and 250 μg/kg/day significantly decreased the acrosome reaction which is an indicator of fertilizing ability of the sperm in mice (148). These studies aimed at evaluating the impacts of BPA on male reproductive health and consistently indicated that exposure to low doses of BPA across all developmental stages affected sperm production and fertilizing quality. Although there were differences in exposure windows, period of time, and species investigated, effects unanimously noticed included reduced sperm number, stimulation of sperm apoptosis and oxidative stress (72, 136, 141–148). A description of the effect of bisphenols on germ cells is shown in Figure 4.

### Bisphenols Are Related to Transgenerational Male Reproductive Health Disorders

Bisphenols are both genotoxic and epigenotoxic (149). The transgenerational inheritance of the effects of endocrine disruptors has been demonstrated in a series of studies (135, 137, 147, 149, 150). A recently published study demonstrated that paternal exposure to BPA during spermatogenesis markedly modified sperm genetic materials and F1 embryo (149). Our previous study indicated that effects of BPA were vertically transferred to sperm of F1 mice after gestational exposure (143). In another study (137), gestational exposure to BPA caused functional and proteomic modifications in F1 capacitated spermatozoa of adult mice. Epigenetic mechanisms are believed to be responsible for these transgenerational effects (151). Epigenetic modification in spermatozoa and other testicular cells could occur via histone modifications, DNA methylation, and noncoding RNAs (151, 152). Recently, it was discovered that the impacts of BPA could be transgenerational and multigenerational via DNA methylation (141). During DNA methylation, a methyl group is added to the cytosine base within a CpG dinucleotide, and methylation is always connected to transcription subjugation (153). Histone modification involves changing the chromatin organization by substituting DNA interaction with other histones, thereby accelerating or reducing transcription chances (154). Noncoding RNAs, including small and long, are involved in chromatin function and the modulation of gene expression through gene silencing or activation (155, 156). Global hypomethylation in human spermatozoa (157) and zebrafish testes have been reported to be caused by BPA (157, 158). In the same manner, BPA has been implicated in hypermethylation of mouse spermatocytes (159). A decrease in histone acetylation in rat testes was observed following long-term exposure to a low concentration of BPA (160), while increase in histone acetylation in zebrafish testes accompanied exposure to high dose of BPA (161). These reports indicate that bisphenols, especially BPA, threaten the reproductive health of direct contact males and successive generations.

### Action Mechanisms of Bisphenols on Germ Cells

BPA and its derivatives exert their effects on different cells and tissues via varying mechanisms and signaling pathways. The effect of exposure to BPA is instant and prompt although observed consequence on gene expression could be delayed by action of the nuclear hormone receptor (36, 162). The mechanism involved in the action of bisphenols on varying cell types is becoming clearer based on recent research findings. The quality and features of receptors implicated in the onset of effect signaling sometimes differ from one cell to the other but are usually associated with those of nuclear hormone receptor-like protein (36). It is generally known that bisphenols possess estrogenic and antiandrogens properties competent of interfering with hypothalamic-pituitary-gonadal axis, especially BPA (11, 32, 52, 163). The structural characteristics of BPA aid its capability to bind with both estrogen receptor types (ERα and ERβ) (17, 164, 165). Germ cells of all developmental stages of rodents and man are known to express ERα and ERβ. As a selective ER modulator, BPA can behave as estrogen agonist or antagonist depending on cell type (166). The activities of bisphenols via estrogen receptors is supported by several studies (36, 167, 168). Through binding to GPER/GPR30, BPA can produce rapid and impactful effects in germ cells (90, 169, 170). Moreover, it was recently reported that IFNβ-XAF1-XIAP pathways mediated BPA induced male germ cell apoptosis in adult mice (125). The multiple pathways involved in the effect of bisphenols, especially BPA, on male germ cells may be why it affects both neonatal and adult rat germ cells, while significant effects of BPA were mainly observed in the Sertoli cells of neonatal rats in most studies.

## Bisphenols Effect on Peritubular Myoid Cells

Peritubular myoid cells are smooth muscle cells that surround the seminiferous tubule in the testis. They are contractile cells which propel spermatozoa to the caput portion of the epididymis (171, 172). Earlier finding also indicated that testosterone-regulated glial cell line-derived neurotrophic factor (GDNF) expression by peritubular myoid cells contributes to the maintenance of spermatogonial stem cell (172). They secrete components of the basement membrane such as fibronectin, collagens, proteoglycans, and entactin (173). In addition, communication between the peritubular myoid and the Sertoli cells is required for the formation of basal lamina during postnatal development (174).

Meanwhile, it has been reported that gestational exposure of female ICR mice to 100 nmol/l per day from gestational day 0 to the end of lactation resulted in apoptosis and mitochondrial vacuolation of peritubular myoid cells of the male pups (175). In another study, an ultrastructural analysis of the testes of adult monkeys (marmosets) exposed to 12.5 and 25 μg/kgbw/day showed the presence of vacuoles in mitochondria of peritubular myoid cells. These reports suggest BPA could interfere with the sperm transport in exposed males. While the effect of bisphenols on Leydig, Sertoli, germ, and peritubular myoid cells are becoming better understood, the impacts on other testicular cell types such as nerve, blood, and lymphatic endothelial cells have not been studied. Therefore, investigation into the effects of bisphenols on these cells is needed, thus providing a roadmap for future studies.

## Future Perspectives

For decades, the impact of low doses of bisphenols on male reproduction has been disputed. While some studies have reported that the administration of bisphenols at low doses does not impact vital alteration in reproductive qualities, other revealed varying degree of harm they unleash on male fertility. In this review, we have shown that bisphenols represent threats to male reproductive health through their actions on Leydig, Sertoli, and germ cells. However, bisphenols are crucial chemicals in household chemical products and plastics industry and are still being used in the production of consumer products around the world due to difficulties in developing economical and safe alternatives (176). It is worthy of note that some of the evidence reported in animal studies may not have same effects on humans for reasons such as stage of development at exposure, cocktail effect, and difference in metabolic process. Therefore, future research should focus on the clarification of the extrapolation of human risk assessment data from rodents due to varying responses to bisphenols among different strains. Secondly, testes are complex organs housing distinct cells that respond to bisphenols differently via varying mechanisms, therefore, holistic studies that simultaneously evaluate the response of Leydig, Sertoli, and germ cells to the same dosages of bisphenols and mechanisms involved are greatly needed. In addition, other testicular cells such as peritubular cells, macrophages, other immune cells, and vasculature should be included among priorities in the study of effect of bisphenols on testicular cells. Fortunately, some approaches have been suggested toward the suppression of BPA toxicities. The pharmacological inhibition of ERKI/2 could be considered a target for mitigating the effects of bisphenols in testicular cells. In addition, producers in real situation do not strictly adhere to a prescribed quantity or dosage by regulatory bodies because they may not provide the desired quality. While the scientific consensus indicates that at a cellular level, BPA, and its analogs alter testicular cell development and functions at low, environmentally relevant doses, future studies should investigate mitigation to protect human health and the environment.

## Author Contributions

EA and MR wrote the manuscript. M-GP conceived the innovations in the manuscript and edited it. All authors thoroughly revised the manuscript and approved its submission.

## Conflict of Interest

The authors declare that the research was conducted in the absence of any commercial or financial relationships that could be construed as a potential conflict of interest.
